# The progesterone antagonist mifepristone/RU486 blocks the negative effect on life span caused by mating in female Drosophila

**DOI:** 10.18632/aging.100721

**Published:** 2015-01-15

**Authors:** Gary N. Landis, Matthew P. Salomon, Daniel Keroles, Nicholas Brookes, Troy Sekimura, John Tower

**Affiliations:** ^1^ Molecular and Computational Biology Program, Department of Biological Sciences, University of Southern California, Los Angeles, CA 90089-2910 USA

**Keywords:** aging, sexual antagonistic pleiotropy, sexual conflict, life span trade-off

## Abstract

Mating causes decreased life span in female Drosophila. Here we report that mifepristone blocked this effect, yielding life span increases up to +68%. Drug was fed to females after mating, in the absence of males, demonstrating function in females. Mifepristone did not increase life span of virgin females or males. Mifepristone reduced progeny production but did not reduce food intake. High-throughput RNA sequencing was used to identify genes up-regulated or down-regulated upon mating, and where the change was reduced by mifepristone. Five candidate positive regulators of life span were identified, including dosage compensation regulator Unr and three X-linked genes: multi sex combs (PcG gene), Dopamine 2-like receptor and CG14215. The 37 candidate negative genes included neuropeptide CNMamide and several involved in protein mobilization and immune response. The results inform the interpretation of experiments involving mifepristone, and implicate steroid hormone signaling in regulating the trade-off between reproduction and life span.

## INTRODUCTION

Mifepristone (RU486) is a progesterone receptor antagonist and glucocorticoid receptor antagonist with human contraceptive and abortifacient activities, and is in clinical trials for its potential as an anticancer drug [1]. Mifepristone is often used in Drosophila aging research as the trigger for the conditional gene expression system called “Gene-Switch” [2]. “Driver” strains express the engineered transcription factor Gene-Switch, which becomes active only when flies are fed mifepristone. Activated Gene-Switch binds to UAS sites in the promoter of target constructs to yield transgene over-expression. Certain previous studies have included controls for possible effects of mifepristone on life span, typically using the progeny of a driver strain crossed to the non-transgenic control strain *w*[1118]. Either no effect [3, 4], a small negative effect [5, 6], or a small positive effect [7, 8] has been reported. Here we report that mifepristone acts in females to block the negative effect of mating, yielding increased life span for several genotypes, including the popular *w*[1118] control strain and *Elav*-Gene-Switch (*Elav-GS*) driver strain [2]. The results indicate that mifepristone may sometimes lead to inflated estimates of female life span increase upon transgene over-expression using Gene-Switch. Moreover, the results implicate steroid hormone signaling and *X*-chromosome gene expression in regulating the trade-off between reproduction and life span caused by mating in female Drosophila.

## RESULTS

### Mifepristone increases adult life span depending on sex and genotype

**Figure 1:**
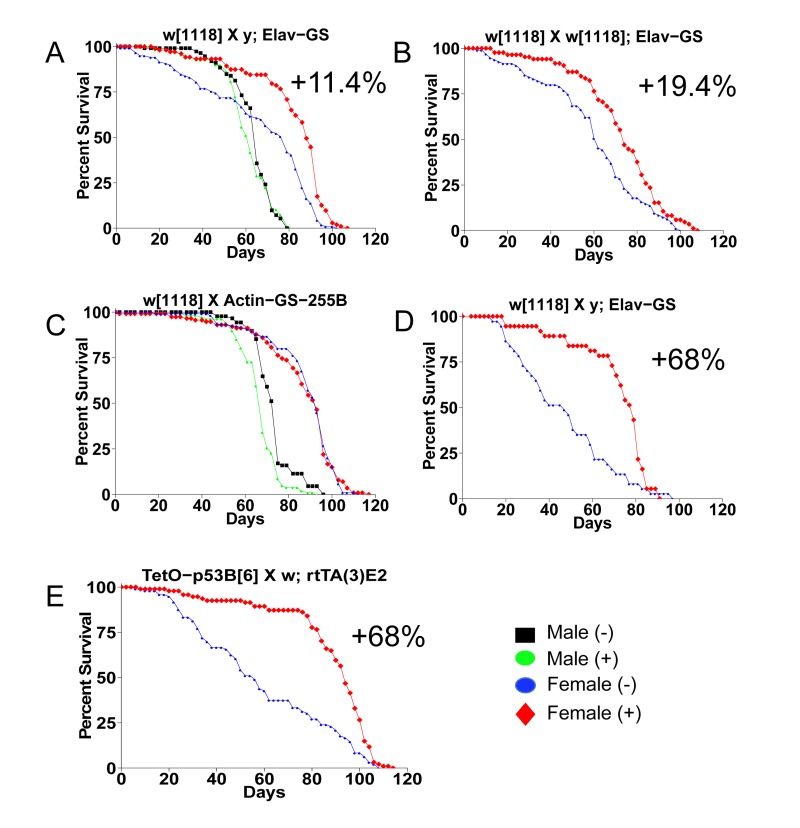
Mifepristone life span increase depends on sex and genotype Survival curves are presented for male and female flies cultured as adults in the presence and absence of 160ug/ml mifepristone, for progeny of the indicated crosses (written in order male x female). (**A**) Control *w*[1118] strain crossed to *y; Elav-GS*. (**B**) Control *w*[1118] crossed to the backcrossed strain *w*[1118]*; Elav-GS*. (**C**) Control *w*[1118] strain crossed to *Actin-GS-255B*. (**D**) Control *w*[1118] strain crossed to *y; Elav-GS*. (**E**) Tet-on system strain *p53B*[6] crossed to *w; rtTA(3)E2*. (**A, C**) Flies were allowed to eclose for 48 hours prior to sorting. (**B, E**) Flies were allowed to eclose for 48 hours and then allowed to mate to siblings for an additional 48 hours prior to sorting. (**D**) Females were collected as virgins over 24 hours, then mated to *w*[1118] males for two days. Statistical summary in Table 1.

**Figure 2:**
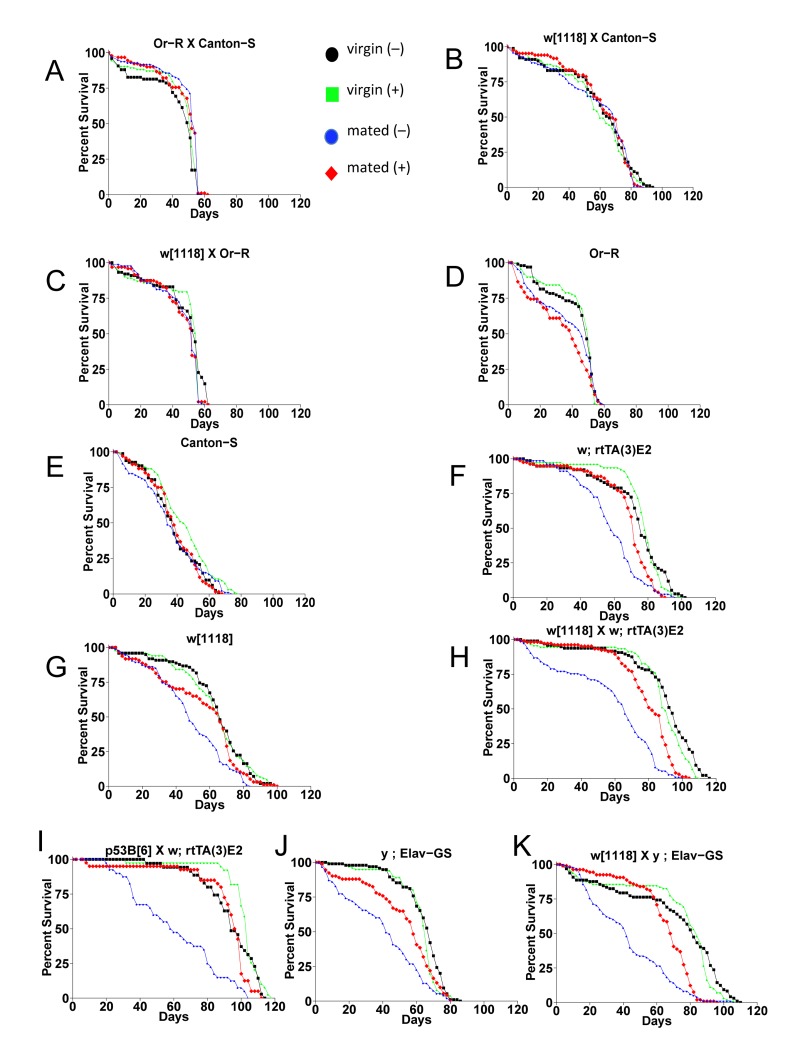
The effect of mating and mifepristone on female life span in multiple genotypes Female progeny from the indicated crosses were collected as virgins over 24 hours, then half of the virgins were immediately mated to *w*[1118] males at a ratio of 1:1 for 4 days. Males were then separated from the mated females, and the virgins and mated females were maintained on media in presence or absence of 160ug/ml drug. Statistical summary in Table 1.

**Figure 3:**
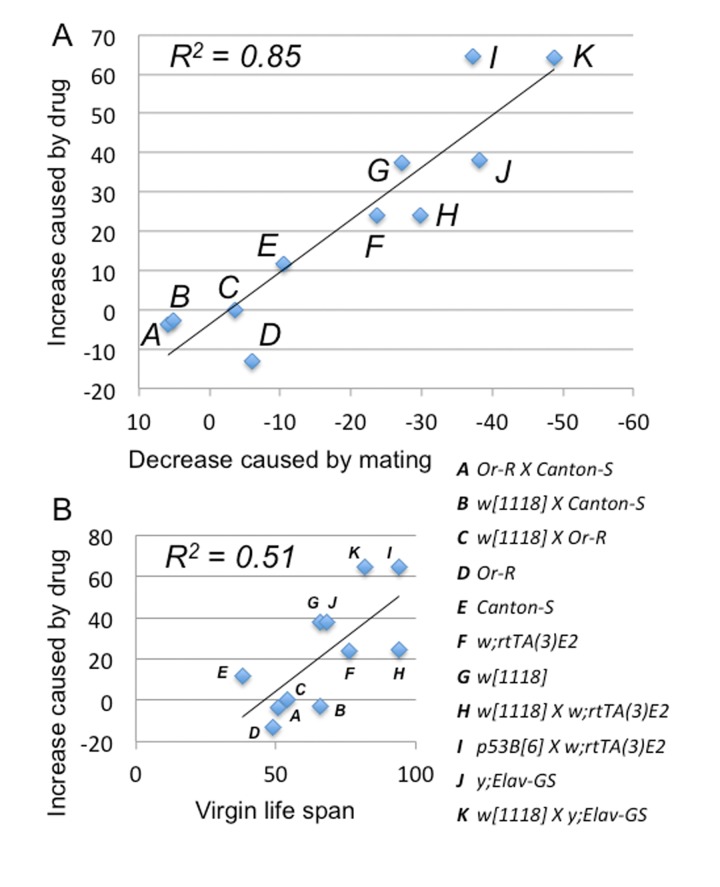
Mifepristone prevents the life span decrease caused by mating Strains and crosses used to generate the 11 female genotypes are indicated (*A-K*; life span curves shown in Figure 2; statistical summary in Table 1). (**A**) The percent decrease in female life span caused by mating (X-axis) is compared to the percent increase in life span caused by mifepristone in mated females (Y-axis). (**B**) The median life span of virgin females (X-axis) is compared to the percent increase in life span caused by mifepristone in mated females (Y-axis).

The first life span assays were conducted using a common approach to generate the age-synchronized cohorts. Flies were allowed to eclose in culture bottles for a period of 48 hours and then males and females were separated. These conditions are expected to result in most, but not necessarily all, flies having mated at least once [4]. The *w*[1118] control strain was crossed to the Gene-Switch system driver strain *Elav-GS*, and feeding the drug mifepristone to the progeny produced no change in life span in males, and an increase in female life span of 11.4% (Figure 1A; replicate experiments and statistical analyses in Table 1). The *Elav-GS* line was backcrossed to the *w*[1118] strain for 9 generations and then re-tested by crossing to *w*[1118], and feeding drug to the female progeny produced a life span increase of 19.4% (Figure 1B). In contrast, the progeny of crosses of *w*[1118] to the Gene-Switch driver strain *Actin-GS-255B* showed no increase in life span with drug in either sex (Figure 1C; Table 1). A small decrease was observed in males in this experiment, however negative effect in males was not observed with other genotypes (Table 1). These results indicated that mifepristone causes a life span increase in females but not in males, and for crosses involving some Gene-Switch driver strains but not all. When longer mating times were employed mifepristone feeding produced larger increases in female life span. The *w*[1118] control strain was crossed to *Elav-GS* and the progeny females were collected as virgins over 24 hours, and then mated to young mature *w*[1118] males at a ratio of 1:1. Females mated for two days had a +68% increase in life span caused by mifepristone (Figure 1D; Table 1) and females mated for four days had a +64% increase caused by mifepristone (Figure 2K; Figure 3; Table 1). An increase in female life span of +68% was caused by mifepristone in the progeny of a cross of two unrelated transgenic laboratory strains (Figure 1E; Table 1), and increases were also observed using non-transgenic *w*[1118] flies (Figure 2G; Figure 3; Table 1), demonstrating that the Gene-Switch transcription factor is not required.

**Table 1:** Life span statistical summary VM=virgin male, M=mated male, V=virgin female, F=mated female. Mating is 4 days unless otherwise noted. Crosses in order: males X virgin females. (+) = 160ug/ml

**Figure 1**									
Genotype	Sex	RU486	n	Mean(SD)	Median	90%	Δ Mean %	Δ Median %	(p)
w[1118] X y; Elav-GS	M	-	112	63 (11)	65	72			
	M	+	108	61 (11)	62	75	−3.88	−4.62	0.3325
w[1118] X y; Elav-GS	F	-	117	66 (26)	79	93			
	F	+	103	82 (19)	88	97	24.5	11.4	1.60E-07
w[1118] X w[1118]; Elav	F	−	95	60 (23)	62	88			
	F	+	85	72 (20)	74	93	21.7	19.4	0.0009
w[1118] X 255B-GS	M	-	88	73 (9)	75	89			
	M	+	106	66 (11)	68	75	−9.31	−9.33	4.53E-05
w[1118] X 255B-GS	F	-	105	88 (17)	93	103			
	F	+	114	86 (19)	93	103	−1.96	0	0.8888
w[1118] X y; Elav-GS	F	-	37	47 (22)	47	77			
	F	+	37	71 (18)	79	85	51.9	68.1	0.0002
p53B[6] X w; rtTA(3)E2	F	-	96	59 (28)	56	98			
	F	+	94	88 (21)	94	106	49.1	67.9	2.63E-08
**Figures 2,3**									
Genotype	Sex	RU486	n	Mean(SD)	Median	90%	ΔMean %	Δ Median %	(p)
y; Elav-GS (J)	V	-	95	64 (13)	68	76			
	V	+	101	62 (13)	66	72	−2.85	−2.94	0.0443
y; Elav-GS (J)	F	-	93	41 (22)	42	68			
	F	+	91	53 (21)	58	74	29.7	38.1	0.0020
w[1118] X w;rtTA(3)E2 (H)	V	-	96	89 (22)	94	108			
	V	+	92	87 (21)	90	106	−2.02	−4.26	0.0284
w[1118] X w;rtTA(3)E2 (H)	F	-	114	57 (27)	66	84			
	F	+	104	78 (18)	82	94	36.1	24.2	2.24E-09
w; rtTA(3)E2 (F)	V	-	76	72 (21)	76	94			
	V	+	78	77 (15)	78	88	5.98	2.63	0.8912
w; rtTA(3)E2 (F)	F	-	80	58 (18)	58	78			
	F	+	79	68 (18)	72	84	17	24.1	0.0013
w[1118] X y;Elav-GS (K)	V	-	97	71 (31)	82	100			
	V	+	98	74 (27)	84	95	3.58	2.44	0.2178
w[1118] X y;Elav-GS (K)	F	-	98	43 (23)	42	75			
	F	+	106	65 (19)	69	82	49.9	64.3	9.21E-07
w[1118] (G)	V	-	98	64 (20)	66	84			
	V	+	102	63 (20)	66	88	−1.06	0	0.9950
w[1118] (G)	F	-	95	49 (21)	48	77			
	F	+	97	56 (24)	66	79	14.6	37.5	0.0043
p53B[6] X w; rtTA(3)E2 (I)	V	-	35	94 (17)	94	112			
	V	+	39	102 (13)	104	112	8.48	10.6	0.0951
p53B[6] X w; rtTA(3)E2 (I)	F	-	40	61 (26)	59	98			
	F	+	40	91 (21)	97	106	49.6	64.4	1.04E-06
Or-R (D)	V	-	97	43 (15)	49	54			
	V	+	90	44 (14)	51	54	2.71	4.08	0.6764
Or-R (D)	F	-	91	37 (18)	46	54			
	F	+	82	34 (18)	40	54	−9.71	−13	0.1175
Canton-S (E)	V	-	82	38 (16)	38	58			
	V	+	94	44 (17)	45	67	15.3	18.4	0.0118
Canton-S (E)	F	-	86	36 (19)	34	64			
	F	+	68	38 (15)	38	56	4.83	11.8	0.6997
w[1118] X Or-R (C)	V	-	88	47 (16)	54	62			
	V	+	93	47 (16)	56	56	−0.02	3.7	0.3270
w[1118] X Or-R (C)	F	-	91	46 (14)	52	56			
	F	+	95	45 (14)	52	56	−0.93	0	0.9303
Or-R X Canton-S (A)	V	-	75	42 (17)	51	56			
	V	+	94	45 (15)	51	56	7.84	0	0.3466
Or-R X Canton-S (A)	F	-	98	48 (13)	54	56			
	F	+	90	47 (14)	52	56	−2.78	−3.7	0.7202
w[1118] X Canton-S (B)	V	-	89	59 (23)	66	84			
	V	+	81	58 (22)	60	82	−2.22	−9.09	0.5404
w[1118] X Canton-S (B)	F	-	97	58 (24)	70	80			
	F	+	85	61 (20)	68	80	5.68	−2.86	0.9908
**Figure 4**									
Genotype	Sex	RU486	n	Mean(SD)	Median	90%	ΔMean %	Δ Median %	(p)
**Figure 4A**									
p53B[6] X w; rtTA(3)E2	VM	-	69	96 (13)	101	109			
	VM	5	58	92 (15)	97	105	−4.68	−3.96	0.0611
	VM	160	54	96 (18)	101	109	−0.918	0	0.4943
	VM	640	54	34 (11)	31	39	−64.6	−69.3	0.0000
**Figure 4B**									
p53B[6] X w; rtTA(3)E2	V	-	57	94 (26)	103	113			
	V	160	58	97 (19)	103	108	3.38	0	1.0000
	V	640	61	44 (12)	47	55	−53	−54.4	0.0000
**Figure 4C**									
p53B[6] X w; rtTA(3)E2	M	-	46	92 (08)	96	99			
	M	5	51	90 (14)	91	109	−1.99	−5.21	0.7930
	M	160	59	95 (15)	99	105	2.93	3.13	0.0071
	M	640	55	49 (21)	39	79	−47.2	−59.4	0.0000
**Figure 4D**									
p53B[6] X w; rtTA(3)E2	F	-	55	70 (28)	73	102			
	F	160	57	87 (24)	95	108	24.8	30.1	0.0009
	F	640	58	45 (14)	45	67	−35.1	−38.4	1.20E-10
**Figure 4E**									
Treatment	Sex	RU486	n	Mean(SD)	Median	90%	ΔMean %	Δ Median %	(p)
0 ug/ml	F	-	40	60 (31)	67	95			
2.5 ug/ml	F	+	41	58 (30)	63	91	−3.45	−5.97	0.7737
5.0 ug/ml	F	+	36	66 (31)	67	109	8.81	0	0.1100
10 ug/ml	F	+	37	76 (28)	81	108	25.3	20.9	0.0110
20 ug/ml	F	+	40	78 (33)	94	109	29.2	40.3	0.0005
40 ug/ml	F	+	39	81 (26)	89	111	34.2	32.8	0.0006
80 ug/ml	F	+	38	77 (32)	93	103	27.1	38.8	0.0132
160 ug/ml	F	+	38	93 (12)	95	107	54.8	41.8	1.05E+05
**Figure 6**									
**Figure 6A**									
Treatment	Sex	RU486	n	Mean(SD)	Median	90%	ΔMean %	Δ Median %	(p)
(−)	F	-	96	40 (17)	39	64			
(+)	F	+	100	51 (13)	50	66	25.9	28.2	0.0020
Day 20 off	F	+	59	50 (16)	52	70	23.3	33.3	0.0128
Day 30 off	F	+	58	50 (14)	50	67	24.1	28.2	0.0182
Day 20 on	F	+	62	41 (17)	38	66	2.53	−2.56	0.6411
Day 30 on	F	+	54	43 (12)	43	54	7.14	10.3	0.9018
Day 40 on	F	+	61	44 (16)	44	64	9.67	12.8	0.2646
**Figure 6B,C and additional data**									
Genotype	Sex	RU486	n	Mean(SD)	Median	90%	ΔMean %	Δ Median %	(p)
w[1118] x PGC1α-III	M	-	112	71 (15)	77	79			
	M	+	107	69 (18)	75	77	−1.97	−2.6	0.3528
	F	-	116	78 (23)	87	96			
	F	+	103	82 (18)	87	97	5.77	0	0.4981
5961-GS x PGC1α-III	M	-	96	74 (13)	77	88			
	M	+	100	75 (9)	77	83	0.98	0	0.4805
	F	-	92	75 (12)	77	87			
	F	+	94	76 (9)	77	83	0.67	0	0.3127
B3B-GS x PGC1α-III	M	-	118	72 (13)	76	81			
	M	+	116	69 (14)	75	77	−3.66	−1.32	0.0801
	F	-	111	80 (16)	83	95			
	F	+	114	78 (15)	80	91	−2.95	−3.61	0.0137
255B-GS x PGC1α-III	M	-	116	74 (15)	77	88			
	M	+	107	67 (12)	67	77	−9.48	−12.99	3.78E-09
	F	-	98	83 (21)	91	99			
	F	+	91	82 (15)	83	95	−0.71	−8.79	0.0101
y;Elav-GS x PGC1α-III	M	-	107	71 (14)	73	82			
	M	+	99	68 (10)	69	77	−4.36	−5.48	0.0005
	F	-	108	73 (18)	78	88			
	F	+	123	63 (18)	68	79	4.97	3.85	0.1645
									
Cohort 1									
Genotype	Sex	RU486	n	Mean(SD)	Median	90%	ΔMean %	ΔMedian %	(p)
w[1118] x PGC1α-III	F	-	122	57 (20)	64	78			
	F	+	120	55 (19)	60	76	−3.41	−6.25	0.1403
	F	5	121	65 (20)	72	84	13.6	12.5	0.0006
PGC1α-III X 5961-GS	F	-	113	72 (21)	78	90			
	F	+	123	72 (19)	76	92	−0.92	−2.56	0.5778
	F	5	120	79 (20)	86	90	9.07	10.3	0.0174
5961-GS X PGC1α-III	F	-	124	82 (18)	88	94			
	F	+	118	72 (19)	76	88	−11.8	−13.6	1.56E-10
	F	5	124	80 (16)	84	92	−1.68	−4.55	0.02814
Cohort 2									
Genotype	Sex	RU486	n	Mean(SD)	Median	90%	ΔMean %	Δ Median %	(p)
w[1118] x PGC1α-III	F	-	117	74 (16)	78	90			
	F	+	120	69 (16)	72	84	−7.21	−7.69	0.0001
	F	5	119	70 (16)	72	84	−5.86	−7.69	0.0017
PGC1α-III X 5961-GS	F	-	121	83 (13)	88	92			
	F	+	119	76 (18)	82	90	−8.34	−6.82	1.68E-05
	F	5	120	80 (15)	84	92	−3.69	−4.54	0.2951
5961-GS X PGC1α-III	F	-	124	77 (16)	80	92			
	F	+	124	81 (20)	88	92	6.03	10	0.0004
	F	5	123	81 (13)	86	92	6.21	7.5	0.0476

### Mating is required for mifepristone to increase female life span

**Figure 4:**
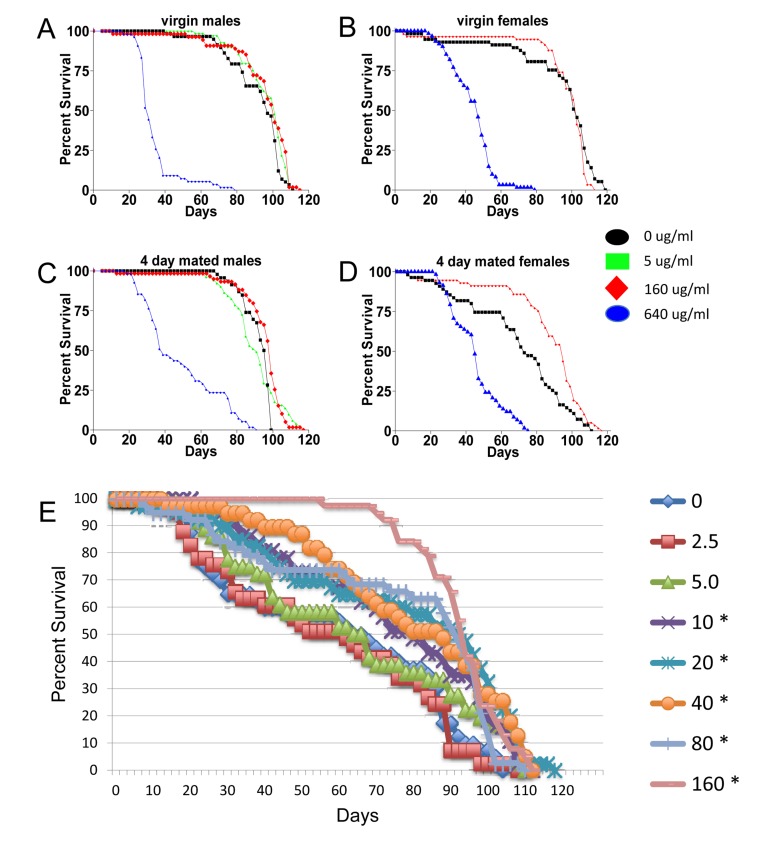
Characterization of mifepristone effects (**A-D**) Effect of mifepristone on virgin and mated male and female life span. The *p53B*[6] strain males were crossed to *w; rtTA(3)E2* strain females. The male and female progeny were collected as virgins over 24 hours, then mated to *w*[1118] virgins and males, respectively, at a ratio of 1:1 for 4 days. After the 4 days mating, mates were removed and the flies were maintained on media in presence or absence of the indicated concentration of drug. (**A**) Virgin males. (**B**) Virgin females. (**C**) Males mated for 4 days. (**D**) Females mated for 4 days. (**E**) Dose-response. The control *w*[1118] strain was crossed to *y; Elav-GS* strain and female progeny were assayed for life span in presence and absence of drug. The concentration of drug in the media was titrated in a range from 2.5ug/ml to 160ug/ml, as indicated. Flies were allowed to eclose for 48 hours and then mated to siblings for an additional 48 hours prior to sorting. Asterisks indicate statistically significant difference (p < 0.05) between drug treated and no-drug control as determined using log rank tests.

**Figure 5:**
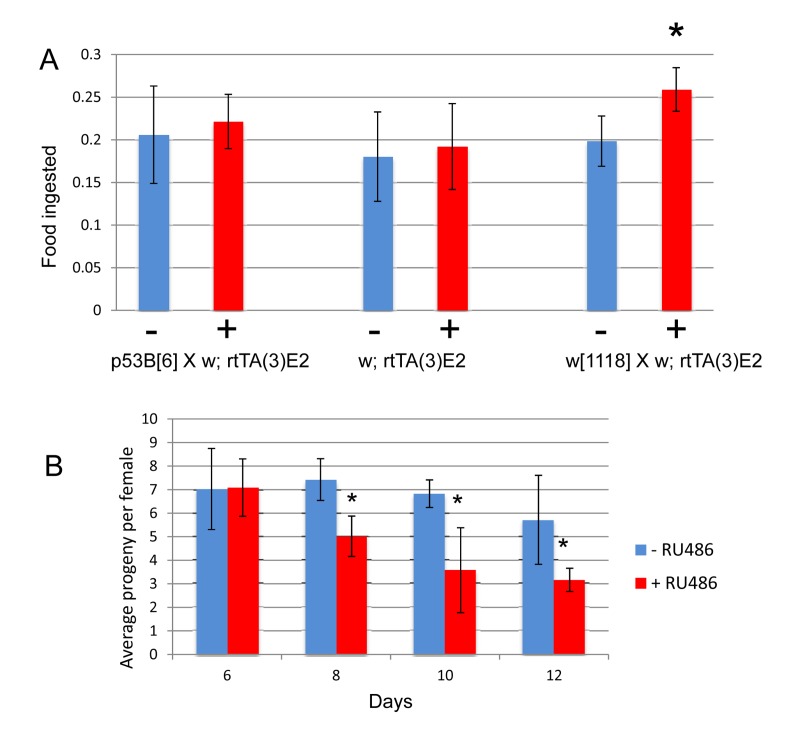
Effect of mifepristone on feeding and progeny production (**A**) Feeding assay. The indicated crosses were conducted and virgin females were collected over 24 hours and then mated to *w*[1118] males at a ratio of 1:1 for 4 days. Mated females were then separated from the males and cultured as adults in the presence and absence of 160ug/ml drug for 20 days. Feeding was estimated using dye uptake assay, using 6 biological replicates per sample, and 5 flies for each replicate. Plus-drug was compared to minus-drug using unpaired, two-sided t-tests, and statistically significant differences (p < 0.05) are indicated with asterisk. (**B**) Progeny production. The strain *p53B*[6] was crossed to *rtTA(3)E2* and the female progeny were collected as virgins over 24 hours, and then mated to *w*[1118] males at a ratio of 1:1 for 4 days. Mated females were then separated from the males and cultured as adults in the presence and absence of 160ug/ml drug. 5 replicate vials of 20 females per vial. Number offspring per female is plotted as average +/− SD. Plus-drug compared to minus-drug using unpaired, two-sided t-tests, statistically significant difference (p < 0.05) indicated with asterisk.

Mifepristone had no consistent effect on the life span of virgin females (Figure 2; Figure 4B; Table 1), virgin males or mated males (Figure 4A, C; Table 1). In these experiments mifepristone is being used at 160ug/ml. Most previously published studies have used mifepristone at concentrations ranging from 25ug/ml to 200ug/ml. Titration of mifepristone revealed a dose-response for life span increase, and significant effects were observed with concentrations as low as 10ug/ml (Figure 4E; Table 1). Concentrations of 640ug/ml were toxic, and reduced life span in males, virgins and mated females (Figure 4A-D; Table 1). To address the possibility that mifepristone might make the food unpalatable, thereby causing a dietary restriction effect, food consumption was assayed in the presence and absence of mifepristone. This was done using several geno-types, including the genotype exhibiting the largest life span increase due to mifepristone (as shown in Figure 1E), and no decrease in food consumption was detected (Figure 5A), indicating that the flies are not diet restricted. Indeed in some experiments a small increase in food consumption was detected (Figure 5A). In addition to reducing life span, mating stimulates progeny production by female Drosophila [9]. Mifepristone feeding significantly reduced progeny production by the mated females (Figure 5B), consistent with the idea that mifepristone inhibits the physiological changes normally caused by mating.

### Mifepristone blocks the negative effect of mating on life span

**Figure 6:**
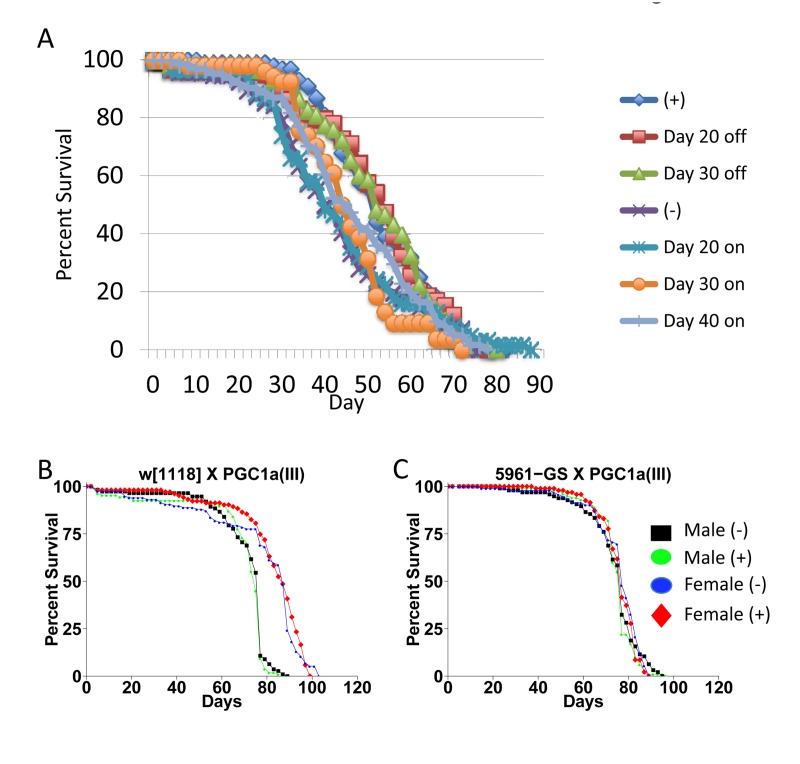
Critical period for drug treatment and effect of dPGC-1 over-expression (**A**) Critical period for drug treatment. Strain *p53B*[6] was crossed to *w; rtTA(3)E2*. Female progeny were collected as virgins over 24 hours, then immediately mated to *w*[1118] males at a ratio of 1:1. The male and female flies were then maintained together on media in presence or absence of 160ug/ml drug for the indicated periods. Males were replaced with young (1-2 weeks of age) *w*[1118] males at day 50. (**B**, **C**) Effect of *dPGC-1* over-expression. The indicated crosses were conducted and progeny flies were allowed to eclose for 48 hours. Flies were then transferred to fresh media and allowed to mate to siblings for an additional 48 hours prior to sorting. Male and female flies were maintained in presence and absence of 160ug/ml mifepristone, as indicated. (**B**) The *w*[1118] control strain was crossed to the *PGC1a(III)* strain. (**C**) The *5961*-Gene-Switch driver strain was crossed to the *PGC1a(III)* strain. Statistical summary for these experiments and additional experiments is presented in Table 1.

The negative effect of mating on female life span was determined by comparing virgin and mated female life spans for 11 common genotypes. The change in life span varied from no effect to –50% (Figure 3A, X-axis; life span curves in Figure 2; statistical summary in Table 1). The increase in life span caused by mifepristone was proportional to the decrease in life span caused by mating, and ranged from no effect to +64% (Figure 3A, Y-axis; Table 1). These results support the conclusion that mifepristone increases female life span by preventing the negative effect of mating. The effect of both mating and mifepristone tended to be greater in genotypes with longer starting life spans (Figure 3B; Table 1). Feeding of mifepristone for the first 20 days of adulthood was sufficient to increase life span of mated females, whereas feeding of mifepristone from day 20 onwards had no detectable effect (Figure 6A; Table 1), suggesting a critical period in young adulthood.

### Transcriptome analysis reveals regulatory effects of mifepristone

**Figure 7:**
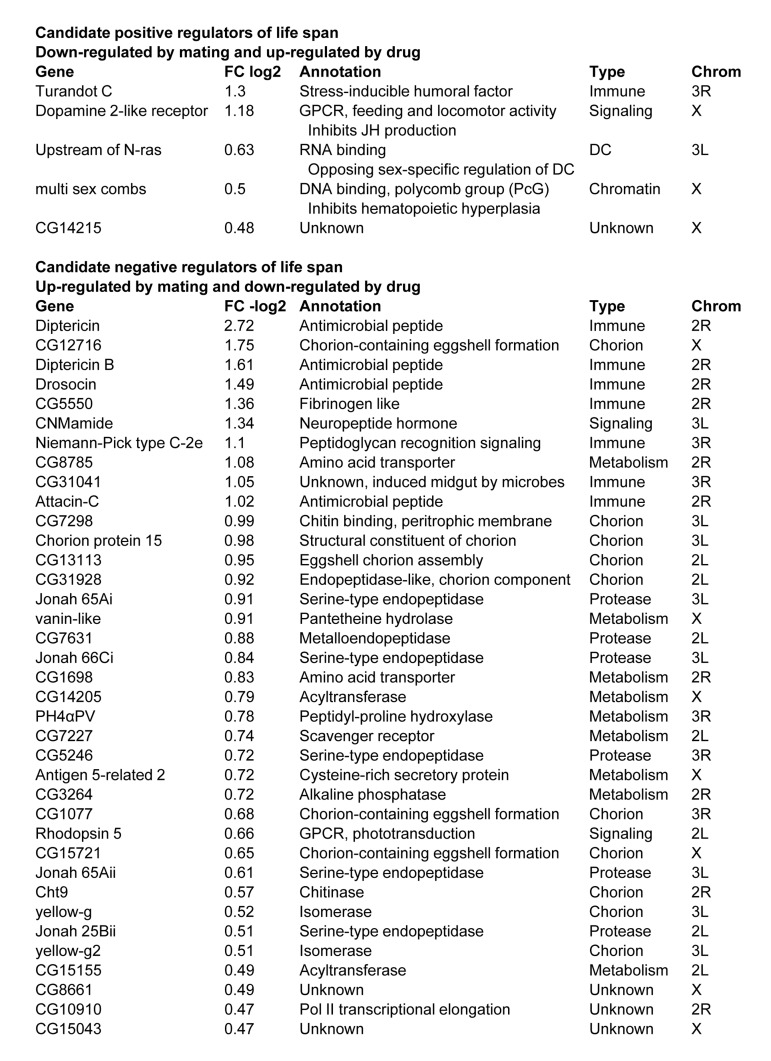
Gene expression changes associated with female life span The fold change (FC) is presented for the effect of mifepristone in mated females. Additional details in Supplemental Table S1.

Patterns of gene expression were analyzed in males, virgin females and mated females in the presence and absence of mifepristone treatment. In mated females mifepristone uniquely increased the expression of 30 genes, and an additional 8 genes were increased to a greater extent than in virgins or in males (Supplemental Table S1). Many of these genes are implicated in steroid metabolism and detoxification, including several cytochrome p450 genes and transferases. Five of these genes were also reduced upon mating in females, and were therefore positively correlated with life span under all conditions (Figure 7). In turn, in mated females, mifepristone uniquely decreased the expression of 40 genes, and an additional 8 genes were decreased to a greater extent than in virgin females or in males (Suppl. Table S1). This group contained many immune function genes and oogenesis genes, consistent with the fact that mifepristone decreases progeny production. The majority of these (37 genes) were also induced by mating in females and were therefore negati-vely correlated with life span under all conditions (Figure 7).

### Gene-switch system over-expression of *dPGC-1* in adult flies

Previous studies reported +17% increase in female life span when the Gene-Switch driver strain *5961-Gene-Switch* was used to drive expression of *dPGC-1* in gut tissue [8]. Using the same strains, as well as additional drivers, we found no consistent effect of *dPGC-1* over-expression in adult flies, using both low (5ug/ml) and high (160ug/ml) concentrations of drug (Figure 6B,C; Table 1).

### Mifepristone is maternal-effect lethal to embryos bearing a Gene-Switch transgene

**Figure 8:**
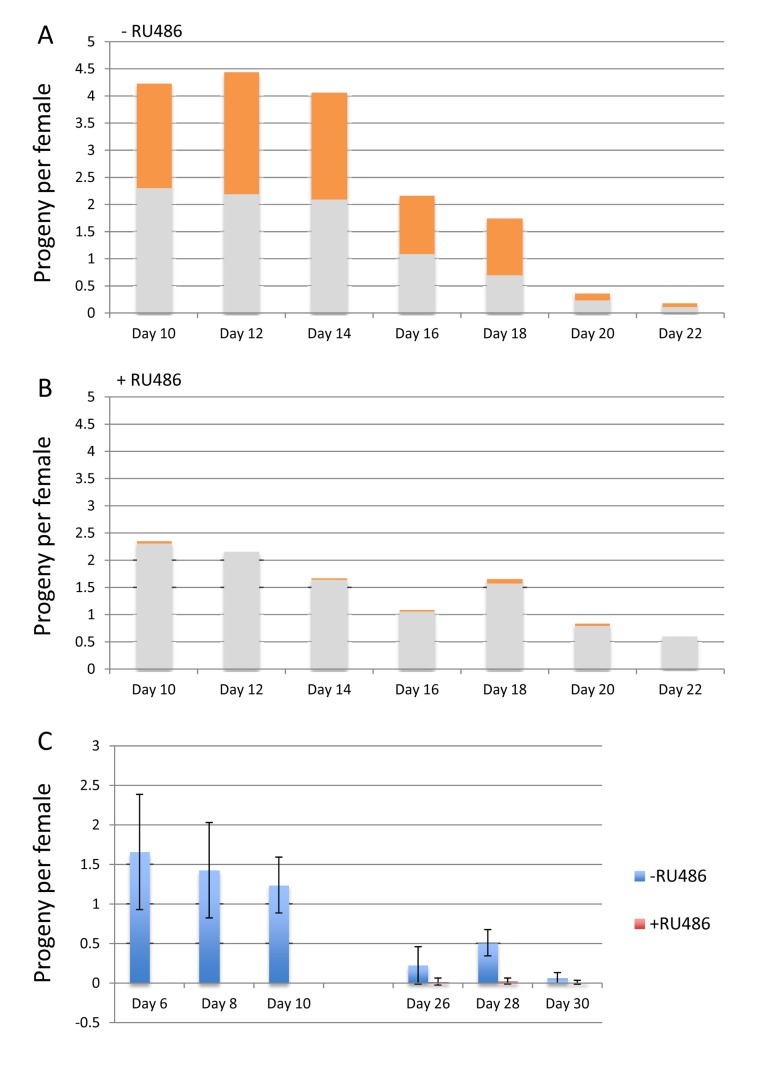
Mifepristone is maternal-effect lethal to embryos expressing Gene-Switch (**A, B**) The *w*[1118] control strain was crossed to *Elav-GS*, and the progeny females were collected as virgins over 24 hours, and then mated to *w*[1118] males at a ration of 1:1 for 4 days. The females were separated from the males and maintained on food in the presence and absence of 160ug/ml mifepristone. The number of adult offspring was quantified for the 2-day intervals, for flies passaged in the presence (**A**) and absence (**B**) of 160ug/ml drug, as indicated. 5 replicate vials of 20 females per vial were used, and the average number of offspring per female per day is plotted as bar graphs. The fraction of the flies bearing the *Elav-GS* transgene is indicated in orange color for each bar. (**C**) The *Elav-GS* strain was crossed to itself and the progeny females were collected as virgins over 24 hours, and then mated to *w*[1118] males at a ration of 1:1 for 4 days. The females were separated from the males and maintained on food in the presence and absence of 160ug/ml mifepristone. The number of adult offspring was quantified for the 2-day intervals, for flies passaged in the presence and absence of 160ug/ml drug, as indicated. 5 replicate vials of 20 females per vial were used, and the average number of offspring per female per day is plotted as bar graphs; error bars indicate standard deviation.

Finally, mifepristone was found to have additional effects relevant to the use of the Gene-Switch system. Specifically, feeding mifepristone to the mother was lethal to embryos containing an *Elav-GS* transgene, even in the absence of any target construct. The *w*[1118] control strain was crossed to *Elav-GS*, and the progeny females were mated to *w*[1118] males and then maintained on food in the presence and absence of 160ug/ml mifepristone. The progeny from these females will be heterozygous for the *Elav-GS* transgene, which contains the mini-*white*+ marker gene and produces an orange-colored eye. In the absence of mifepristone approximately half the progeny contained the *Elav-GS* transgene as expected (Figure 8A, orange bars). However, in the presence of mifepristone the progeny containing *Elav-GS* were almost entirely absent, indicating lethality (Figure 8B). In these vials approximately half the eggs did not hatch, and these un-hatched eggs turned dark and contained dead, partly-developed embryos (data not shown). To confirm this result, *Elav-GS* was crossed to itself and the female progeny were mated to *w*[1118] males, and then maintained on food in the presence and absence of mifepristone. In this cross all of the progeny will contain the *Elav-GS* transgene, and indeed mifepristone was found to be lethal to virtually all the embryos (Figure 8C). Semi-lethality was also observed for embryos containing one copy of the Gene-Switch driver strain *Actin-GS-255B* (data not shown).

## DISCUSSION

### Mifepristone blocks the effect of mating

In these experiments several common laboratory strains of *Drosophila melanogaster* were employed, and mifepristone feeding was found to increase the life span of mated females, but not of virgin females or males. The negative effect of mating on female life span varied dramatically across genotypes, ranging from no effect to –50%. The life span increase caused by drug was proportional to the negative effect of mating, ranging from no effect to +68%, indicating that mifepristone prevents the negative effect on life span caused by mating. Both mating and mifepristone tended to have greater effect in genotypes with longer starting life spans. We speculate that the effect of mating and mifepristone is masked in the shorter-lived backgrounds because of fixed life span-shortening alleles. The drug-treated females did not have reduced food consumption, arguing against a dietary restriction mechanism for the life span increase. Mating normally causes an increase in female oogenesis [9]. Mifepristone reduced progeny production in mated females, consistent with the idea that mifepristone inhibits the physiological changes normally caused by mating, including the trade-off between reproduction and life span.

### Implications for the interpretation of Gene-Switch experiments

The Gene-Switch system has been used to identify many transgenes that can increase life span when over-expressed. These transgenes include, but are not limited to, ones encoding Drosophila *foxo* [10], *Sir2* [11], *p53*-dominant negative [12], *dPGC-1/spargel* [8], *AMPK* and *Atg1* [6], *PGRP-SC2* [13], *InR*-dominant negative, *Akt1* and *bsk* [14], *dCBS* [15], and RNAi knockdown of *ATPsyn-d* [16]. In each case increased life span was observed preferentially or exclusively in female flies. The results presented here suggest that the female-biased increase in life span often observed using Gene-Switch may sometimes include prevention of the negative effect of mating by the transgene and/or by the mifepristone. One of these transgenes (*dPGC-1*) was over-expressed specifically in adults using our conditions and no consistent effect on life span was observed; however differences in media composition cannot yet be ruled out as a possible cause of the differing results.

The Gene-Switch system has been used to identify a small number of genes that increase life span specifically or preferentially in adult males, including nervous system expression of *Gadd45* [17] and tissue-general over-expression of wild-type *p53* [18]. In addition Gene-Switch has been used to identify several transgenes with negative effects on life span in adult females, including *wingless* [5], *dCBS*-RNAi [15], *EcR*-RNAi [19] and tissue-general over-expression of wild-type *p53* [18]. These results, as well as other male-specific effects such as *foxo* mutation interaction with *p53* [20], should generally not be impacted by the positive effects of mifepristone in females reported here, and the opposing effects of *p53* on life span in males and females is supported by analysis of endogenous *p53* mutations [18]. However, the positive effect on life span observed using the *Elav-GS* driver and adult nervous system-specific over-expression of *p53*-dominant negative [12] and *p53* wild-type [20] might possibly include effects of mifepristone.

One approach that has been used in Drosophila life span studies in an attempt to control genetic background is to backcross the transgenic strains to a common background such as *w*[1118]. However the backcrossed strains are typically observed to still vary in starting life span [6, 8, 13, 14], indicating that genetic variation persists that could potentially produce variation in the effects of mating and mifepristone between control and experimental crosses. For example, here mated females of *w*[1118] had median life span of 48 days and an increase caused by mifepristone of 37.5%. In contrast, mated female progeny of *w*[1118] crossed to the backcrossed strain *w*[1118]*; Elav-GS* had a median life span of 62 days and an increase caused by mifepristone of 19.4% (Table 1). A previous study reported that mifepristone decreased progeny production by −17% in females where the fat-body-specific Gene-Switch driver *S_1_-106-GS* drove expression of UAS-GFP [7]; this decrease might have resulted from the mifepristone, the GFP, and/or a maternal-effect embryonic-lethal interaction between the mifepristone and the Gene-Switch driver, such as was observed here for *Elav-GS*.

### Implications for hormonal signaling in the mating response

Previous studies have shown that the increased egg production and decreased life span in the mated female is due in part to the action of transferred male seminal proteins including sex peptide [9, 21]. The sex peptide receptor is a G-protein-coupled receptor (GPCR), and is located on a small number of neurons in the female reproductive tract that also express the gene *ppk* and the sexual-differentiation genes *dsx* and *fru* [22, 23]. Binding of sex peptide inhibits the activity of these neurons, which project to the brain as well as to octopaminergic neurons in the abdominal ganglion. Mating and sex peptide also cause increased expression of innate immune response genes, including anti-microbial peptide (AMP) genes [24]. The exact mechanism for decreased life span caused by mating and seminal proteins is not yet known. It does not require the actual production of eggs, as decreased life span is still observed upon mating in sterile females [25], however the upstream physiological changes and mobilization of resources normally associated with increased reproduction may be involved.

The fact that mifepristone is a steroid hormone antagonist suggests a possible role for steroid hormone signaling in mediating the negative effect of mating. One possibility is that mifepristone competes with an endogenous hormone for binding to its receptor, and thereby prevents a signal that would otherwise increase reproduction and shorten life span in response to mating. One candidate is the steroid hormone ecdysone that controls adult Drosophila sexual differentiation [26]. Ecdysone signaling has been reported to have both positive and negative effects on female life span [19, 27]. A related possibility is that by inducing the expression of detoxification genes, the mifepristone might indirectly promote the inactivation of endogenous hormones. This idea is consistent with the observation that detoxification genes are up-regulated by other manipulations that increase life span in flies, including MnSOD over-expression [28]. In addition to ecdysone, one candidate hormone is the acyclic sesquiterpenoid called juvenile hormone (JH). JH is required for normal oogenesis and is implicated in negative regulation of adult longevity [29], however we observed little overlap in the genes regulated by JH and mifepristone. Other candidates include octopamine and dopamine [23].

### Transcriptome analysis identifies candidate life span regulators

Transcriptional responses to mating and to sex peptide have been previously examined [30, 31]. Those studies revealed regulation of genes involved in innate immune response, oogenesis (including eggshell/chorion genes), behavior and phototransduction, and examples of each were observed here (Figure 4). The ability of mifepristone to block the negative effect on life span enabled us to further define the genes specifically associated with life span changes. Genes up-regulated by mating and down-regulated by mifepristone are candidate negative regulators of life span. Among these 37 genes were 7 innate immune response genes, consistent with the known ability of mating to induce the innate immune response. This group also included 2 amino acid transporters and 6 proteases, consistent with the mobilization of protein reserves to provision the developing oocyte. Finally, as expected, there were 10 genes involved in creation of the eggshell (chorion). Previous studies show that the negative effect of mating and sex peptide on life span is still observed in females where egg production is blocked by mutations [25]. Those results indicate that the negative effect of mating is not due to the later stages of egg production, such as the deposition of protein into the egg (vitellogenesis) or the synthesis of the eggshell. Instead those results indicate that the negative effect of mating on life span is due to upstream physiological changes, such as the mobilization of protein or other effects. Omitting the eggshell genes the candidates for these upstream negative regulators of life span include the immune response genes, the protein mobilization genes, several additional genes involved in metabolic regulation, and the gene encoding the neuropeptide hormone CNMamide.

Genes down-regulated by mating and up-regulated by mifepristone are candidate positive regulators of life span. Across the genome-wide analysis only 5 genes fulfilled these criteria. These genes included the regulator of *X*-chromosome gene expression *Unr,* and three *X*-linked genes: *multi sex combs* (*mxc*), *dopamine 2-like receptor (Dop2R)*, and *CG14215*. *Dop2R* encodes a dopamine receptor that regulates feeding behavior and inhibits JH production [32, 33]. Consistent with a role for dopamine signaling in life span regulation, the genes *Ddc* and *Catsup* regulate the dopamine biosynthetic pathway, and previous studies show that natural variation in these genes affects both courtship behavior and life span [34, 35]. The *mxc* gene is a polycomb-group (PcG) transcriptional repressor that regulates sexual differentiation and gametogenesis [36]. The gene *Unr* encodes an RNA-binding protein that interacts with *Sex lethal* protein and has opposing sex-specific functions in dosage compensation and the regulation of *X*-chromosome gene expression [37, 38]. *X*-chromosome gene expression is implicated in aging [39], and it will be of interest to further examine this relationship using mated Drosophila.

## MATERIALS AND METHODS

### Drosophila stocks and culture

Drosophila were cultured on a standard agar/dextrose/corn meal/yeast media [3] at 25°C, and adult flies were passaged to fresh media every-other day. Drosophila strains are as previously described [4, 18]. Several additional strains were obtained from the Bloomington Drosophila Stock Center, including the *y*[1] w[*]; P{w[+mC]=elav-Switch.O}GSG301 strain (abbreviated *y; Elav-GS*), and the control strain *w*[1118]. The *w*[1118] strain is the sequenced and isogenized version (*w*[1118]*-iso; 2-iso; 3-iso*) generated by Ashburner et al [40, 41]. The *w*[1118] strain was cured of Wolbachia by three generations culture on doxycycline [42], followed by >3 generations culture in absence of doxycycline prior to use. The strain *w; rtTA(3)E2* was previously described, and contains a single insert of a mini-*white+* marked transgene, in the *w*[1118] background [43]. The strain *p53B*[6] contains a single insertion of the USC1.0 vector, containing the mini-*white+* marker gene and the coding region for Drosophila *p53* isoform B, in the *w*[1118] background [18]. The *PGC1a(III)* strain contains a UAS-based over-expression target construct for *dPGC-1* inserted on the third chromosome (Rera et al 2011). This strain and the *5961*-Gene-Switch driver strain were generously provided by David Walker. The *B3B*-Gene-Switch driver is previously described (Nicholson et al 2008) and produced inducible transgene expression in gut tissue (data not shown). All strains were confirmed to be negative for presence of P element transposase using PCR analysis and transposase-specific primers (data not shown), and were confirmed to be negative for Wolbachia using Wolbachia-specific primers [42]. All ages are expressed as days from eclosion at 25oC, and life span assays were conducted as previously described [4] with the following modifications. Flies were maintained as adults at ~25 flies per vial, with transfer to fresh media every other day. Median, percent change in median, and log rank p value were calculated using R statistical environment. Mifepristone (RU486) (Sigma) was fed to adult flies at a final concentration of 160ug/ml in the food media as previously described [3], and in certain experiments mifepristone concentrations were titrated as indicated. To generate age-synchronized cohorts of flies for use in the life span assays the flies were allowed to eclose in culture bottles for 48 hours prior to sorting of males and females, as previously described [4]. In certain experiments the flies were transferred to new culture bottles and allowed to mate with siblings for an additional 48 hours prior to sorting, as indicated. Finally, in certain experiments virgin females were collected over 24 hours, and mating was then conducted by combining with an equal number of young (1-2 weeks of age) *w*[1118] strain males, for either 2 days or 4 days, as indicated. Details of cohort collection methods and mating times are presented in the figure legends for each experiment.

### Transcriptome analysis

The genotype exhibiting the greatest response to mating and to mifepristone was used (progeny of cross *w*[1118]*; p53[B6] x w*[1118]*; rtTA(3)E2*). Males of strain *w*[1118]*; p53B*[6] were crossed to virgins of *w*[1118]*; rtTA(3)E2* and progeny males and virgins were collected over 48 hours. One half of the virgins were mated to *w*[1118] males at ratio of 1:1 virgins to males for 4 days. Mated females were then separated from the *w*[1118] males. The mated females, males and virgins females were then maintained at approximately 20 flies per vial, on food with and without supplementation with 160ug/ml mifepristone for 12 days. Total fly RNA was isolated from 20 animals per sample. Three replicate samples were generated for each type of flies: males, mated females and virgin females. Sequencing libraries were prepared from total RNA using the KAPA Stranded RNA-Seq Library Preparation Kit for Illumina platforms (KAPA Biosystems). Isolation of mRNA was done using the Dynabeads® mRNA purification kit (Invitrogen Dynal AS, Oslo, Norway), following the Appendix A in the KAPA kit protocol. All 18 libraries were indexed using standard Illumina TruSeq indexes, and sequenced altogether in one lane of a high-output paired-end 100 bp run using an Illumina HiSeq 2500. A second high-output paired-end 100 bp run of all 18 libraries was performed to increase coverage. The library preparation and the sequencing were done at the UPC Genome Core facility at the University of Southern California (Los Angeles, CA). The raw RNA sequence data was processed using Trimmomatic [44] to remove any remaining Illumina adapter sequences and low quality bases from the reads. The processed reads were then mapped to the Ensembl BDGP5.25 build of the *D. melanogaster* (downloaded from the Illumina iGenomes website) reference genome using Tophat (version 2.0.12) [45]. Transcript abundance estimates were performed using Cufflinks (version 2.2.1), Cuffmerge (version 2.2.1) was used to combine data across replicates, and Cuffdiff (version 2.2.1) was used to estimate differential expression as described in [46]. Lists of significantly differentially expressed genes were extracted from the Cuffdiff output using the CummbeRbund (version 2.8.2) package in R/Bioconductor [46]; see Table S2. Sequencing coverage was ~33X per replicate. GEO accession GSE64474.

### Feeding assay

Female progeny from the indicated crosses were collected as virgins over 24 hours and then mated to *w*[1118] males at ratio of 1:1 for two days. The female flies were then separated from the males and cultured on +/−mifepristone food for 12 days. The feeding assay was essentially as previously described [47]. The flies were transferred to food vials containing blue dye (20% v/v, Kroger Brand blue food color), at 6 flies per vial. The flies were allowed to feed on the dyed food for 6 hours, starting at 11AM. Six replicate samples of 5 flies each were used for each treatment. The flies were placed in microtubes and homogenized in 200ul deionized water. Flies maintained on food without dye were used to generate a blank sample. Samples were spun in the microcentrifuge (Eppendorf) at full speed for 5 minutes, and supernatents were transferred to new tubes. An additional 800ul deionized water was added to bring volume to 1 ml. Absorbance was measured at 625nm [48] using the spectro-photometer, and the blank value was subtracted from each. Data is plotted as average +/−SD for the 6 replicates. Plus-drug was compared to minus-drug for each genotype using unpaired, two-sided t-tests, and statistically significant differences (p < 0.05) are indicated by asterisk.

## SUPPLEMENTARY TABLE


